# The effectiveness of daily supplementation with 400 or 800 µg/day folate in reaching protective red blood folate concentrations in non-pregnant women: a randomized trial

**DOI:** 10.1007/s00394-017-1461-8

**Published:** 2017-04-26

**Authors:** Rima Obeid, Christiane Schön, Manfred Wilhelm, Klaus Pietrzik, Stefan Pilz

**Affiliations:** 1 Aarhus Institute of Advanced Studies, University of Aarhus, Høegh-Guldbergs Gade 6B, Building 1632, 8000 Aarhus C, Denmark; 2 BioTeSys GmbH, Schelztorstr. 54-56, 73728 Esslingen, Germany; 3 Department of Mathematics, Natural and Economic Sciences, University of Applied Sciences Ulm, Albert-Einstein-Allee 55, 89081 Ulm, Germany; 4 Department of Nutrition and Food Science, Rheinische Friedrich-Wilhelms University, 53115 Bonn, Germany; 5 Division of Endocrinology and Diabetology, Department of Internal Medicine, Medical University of Graz, Auenbruggerplatz 15, 8036 Graz, Austria

**Keywords:** (6S)-5-CH_3_-H_4_folate-Ca, Folic acid, RBC-folate, Homocysteine, Preconceptional, Neural tube defects, Supplementation

## Abstract

**Purpose:**

Folate required to achieve desirable red blood cell (RBC) folate concentrations within 4–8 weeks pre-pregnancy is not known. We studied the effect of supplementation with 400 or 800 µg/day folate in achieving RBC-folate ≥906 nmol/L.

**Methods:**

Non-pregnant women were randomized to receive multinutrient supplements containing 400 µg/day (*n* = 100) or 800 µg/day (*n* = 101) folate [folic acid and (6S)-5-CH_3_-H_4_folate-Ca (1:1)]. The changes of folate biomarkers were studied after 4 and 8 weeks in the 198 women who returned at least for visit 2.

**Results:**

At baseline, 12 of the 198 participants (6.1%) had RBC-folate <340 nmol/L, but 88% had levels <906 nmol/L. The RBC-folate concentrations increased significantly in the 800 µg/day (mean ± SD = 652 ± 295 at baseline; 928 ± 330 at 4 weeks; and 1218 ± 435 nmol/L at 8 weeks) compared with the 400 µg/day [632 ± 285 at baseline (*p* = 0.578); 805 ± 363 at 4 weeks (*p* < 0.001); 1021 ± 414 nmol/L at 8 weeks (*p* < 0.001)]. The changes of RBC-folate were greater in the 800 µg/day than in the 400 µg/day at any time (changes after 8 weeks: 566 ± 260 vs. 389 ± 229 nmol/L; *p* < 0.001). Significantly more women in the 800 µg group achieved desirable RBC-folate concentrations at 4 weeks (45.5 vs. 31.3%; *p* = 0.041) or 8 weeks (83.8 vs. 54.5%; *p* < 0.001) compared with the 400 µg group. RBC-folate levels below the population median (590 nmol/L) were associated with a reduced response to supplements.

**Conclusions:**

88% of the women had insufficient RBC-folate to prevent birth defects, while 6.1% had deficiency. Women with low RBC-folate were unlikely to achieve desirable levels within 4–8 weeks, unless they receive 800 µg/day. The current supplementation recommendations are not sufficient in countries not applying fortification.

**Trials register:**

The trial was registered at The German Clinical Trials Register: DRKS-ID: DRKS00009770.

**Electronic supplementary material:**

The online version of this article (doi:10.1007/s00394-017-1461-8) contains supplementary material, which is available to authorized users.

## Introduction

Preconceptional supplementation with folate increases maternal folate status and prevents a significant number of births with neural tube defects (NTDs) [1–4], even when women are not folate deficient. Therefore, it is recommended that women planning pregnancy or capable of being pregnant should achieve additional intake of 400 µg/day folic acid through supplements and/or fortified foods at least 1 month before pregnancy [5, 6]. This supplementation recommendation for young women is thus well above the population recommended dietary allowance (RDA) of folate (300-400 µg/day) that intends to prevent severe consequences of folate deficiency such as anaemia [5–7].

Practicing of folate supplementation remains low in general, especially in unplanned pregnancy [8]. For this reason, over 70 countries worldwide implemented mandatory fortification with minor amounts of folic acid. This strategy is not applied in Europe including Germany, where the intake of folate from the diet is not sufficient to achieve optimal folate status and voluntary fortification with folic acid has failed to improve folate status markers [9]. In a country like Germany, approximately 500 births with NTD could be prevented each year if all young women can achieve optimal folate status before conception [10].

The World Health Organization (WHO) has recently recommended an optimal population threshold for red blood cell (RBC)-folate (>906 nmol/L) in non-pregnant women of reproductive age [11]. This threshold is derived from an observational study on RBC-folate and NTD risk [12] in addition to a mathematical modelling of RBC-folate and NTD risk [13]. The minimal threshold of 906 nmol/L has been achieved in countries applying folic acid fortification; while optimizing folate status in populations not exposed to fortification remains a major challenge. Increasing folate status through supplementation depends on folate dose [14–16], baseline folate status [17], and duration of the supplementation. However, the daily folate dose needed to achieve RBC-folate levels ≥906 nmol/L within 4–8 weeks is not well established. The current study investigated the effect of 2 doses of folate (folic acid plus (6S)-5-CH_3_-H_4_folate-Ca, 1:1) in raising RBC-folate concentrations in German women of childbearing age within 4 or 8 weeks. We used 400 or 800 µg/day folate for 8 weeks and studied changes of serum folate, RBC-folate, and plasma total homocysteine (tHcy).

## Subjects and methods

### Study setting, participants and design

This is a randomized open labelled controlled study that was conducted between January and May 2016 at BioTeSys GmbH, Esslingen am Neckar, Germany. The primary outcome of the study was changes in serum vitamin D after supplementation of two different doses of vitamin D provided as multinutrient supplements [18]. The secondary outcomes were changes in folate concentrations (serum and RBC-folate) and plasma tHcy. The present paper is concerned with the secondary outcome markers.

Participating women were randomized to receive either 400 or 800 µg/day folate as oral multinutrient supplements containing 1:1 of folic acid and (6S)-5-methyltetrahydrofolate-Ca [(6S)-5-CH_3_-H_4_folate-Ca]. The randomization was conducted using the software Randist.exe. The supplementation was conducted for 8 weeks starting from the recruitment visit 1 (Study flow chart, Supplemental Fig. 1).

Inclusion criteria were: healthy women, age 18–45 years, and BMI between 17 and 30 kg/m^2^. Exclusion criteria included taking supplements containing folate or vitamin D during the last 2 months, vascular diseases, history of cancer, diabetes, or contraindication of iodine supplementation. Gastrointestinal disorders that affect folate absorption such as Crohn’s disease, celiac disease, colitis ulcerosa, inflammatory bowel disease, and peptic ulcer were also exclusion criteria. Exclusion criteria and medical history were based on self-reported information, but several laboratory markers were measured to ensure the general health condition (i.e., liver markers, creatinine, and blood count). Since vitamin D was the main outcome of the study, an exclusion criterion was traveling to southern countries during the intervention (see study design under DRKS-ID: DRKS00009770).

Announcements in local newspapers and medias were used to invite volunteers. Interested women (*n* = 403) were preliminary screened by a structured telephone interview. Women who were likely to meet the inclusion criteria and none of the exclusion criteria (*n* = 213) were invited to the study centre and all of them attended the screening visit. After the screening visit, 12 women had to be excluded because of at least one of the exclusion criteria. The remaining 201 women were randomized and started the intervention (100 women in the 400 µg/day group and 101 in the 800 µg/day group). One woman in the 400 µg/day group and 2 in the 800 µg/day group did not return for the following visits, 1 woman in the 400 µg/day group and 1 in the 800 µg group did not return for visit 3, thus leaving 98 women in each of the study groups after 8 weeks (Supplemental Fig. 1).

A 3 days food diary protocol including alcohol intake was obtained prior to visit 1 and again 8 weeks later (prior to visit 3). Average intake levels were calculated from both assessment periods.

Participants received study fees as single payment upon completion of the study to enhance motivation and compensate for time and transportation to the study site. The study was conducted according to the ethical principles for medical research involving human subjects stated in Helsinki Declaration. The study protocol was reviewed and approved by the medical ethics commission of the Baden-Württemberg region (approval number: F-2015-102). All participants provided a signed consent to the study.

### Supplement compositions, compliance, and safety

Participants were randomized to receive a daily capsule containing 400 µg folate [folic acid and (6S)-5-CH_3_-H_4_folate-Ca (1:1)] (Elevit^®^ gynvital) or a tablet containing 800 µg folate [folic acid + (6S)-5-CH_3_-H_4_folate-Ca (1:1)] (Femibion^®^ 1). The compositions of the multinutrients preparations are shown in Supplemental Table 1. Compliance with the study supplementation was documented through counting the tablets or capsules at each visit in addition to recording the intake in a study diary. The participants were asked to document any suspected adverse events in the volunteer’s diaries.

### Blood samples and processing

Blood samples were collected at baseline (visit 1), week 4 (visit 2) and week 8 (visit 3). To avoid an acute effect caused by the last supplemental dose on plasma and serum biomarkers, participants were instructed to abstain from the study products 24 h before the blood collection (visits 2 and 3). Blood was collected after ≥10 h fasting into tubes without any anticoagulant and those containing K^+^EDTA or K^+^EDTA plus NaF (for plasma glucose). Serum and K^+^EDTA-whole blood were centrifuged and separated within 30 min of blood collection. For the measurement of whole blood folate, whole blood haemolysates were immediately prepared by diluting whole blood with 0.5% ascorbic acid and incubation for 3 h at ambient temperature. After separation (plasma, serum) or preparation (blood haemolysates), samples were immediately frozen at −70°C until analyses of the biomarkers. Samples from the same participant were measured at the end of the study in the same run. All markers were measured using aliquots that were not thawed before.

### Biochemical assays

Concentrations of serum- and whole blood folate were measured using Chemoluminescence immunoassay (IMMULITE®). The concentrations of RBC-folate (nmol/L) were calculated according to the recommendation of the Centers for Disease Control and Prevention by adjusting for haematocrit and serum folate measured in the same individual https://www.cdc.gov/nchs/data/nhanes/nhanes_03_04/l06_c_met_folates-b12.pdf:

$${\text{RBC-folate }} = \, \left( {{\text{whole blood folate }} \times {\text{ dilution factor}}} \right) \, - \, \left[ {{\text{serum folate }} \times \, \left( { 1- {\text{haematocrit}}} \right)} \right] \, \times { 1}00/{\text{haematocrit}}.$$

The concentrations of tHcy were measured in EDTA plasma using commercially available reagents (Chromsystems Instruments & Chemicals GmbH) and a reversed phase-high pressure liquid chromatography connected to a fluorescence detector. The between-day coefficients of variation (CV%) for the folate assay were 8.8% at 3.6 nmol/L and 5.2% at 24.9 nmol/L. The CVs% for the tHcy assay were ≤8.2% at 10.0 µmol/L and 20.6 µmol/L.

Blood count (in K^+^EDTA whole blood), glucose (in NaF-plasma) and markers of liver and kidney functions (in serum) were measured. Measurements of the routine markers were conducted on the same day of blood collection (visits 1, 2, 3) using automatic analysers (Advia 2120i and Advia 2400).

### Study power and statistical analyses

Folate markers (RBC- and serum folate) were secondary outcomes in this trial. The samples size (*n* = 100 per group) calculation was performed for the primary outcome variable, vitamin D. Based on earlier studies in young women from the same country [14, 19], we predicted a mean [standard deviation (SD)] baseline serum folate to be approximately 18.0 (9.0) nmol/L and that of RBC-folate to be 600 (250) nmol/L. For the priority folate marker, RBC-folate, the differences between the intervention groups were expected to be at least 15% after 4 or 8 weeks [mean difference = 170 (250) nmol/L]. Using different scenarios, we estimated that 100 participants in each group will be sufficient for detecting the differences in RBC-folate between the groups with 0.95 power and α = 0.05.

Data analyses included the 198 women who returned at least for visit 2 (intention-to-treat). For the two subjects stopping the study prior to visit 3, missing data were imputed by LOCF principle. The distributions of the continuous variables were tested using the Shapiro–Wilk test. The following variables showed normal distribution; creatinine, fasting glucose, haemoglobin, and haematocrit, while all folate markers were not normally distributed.

Testing the differences in continuous variables between two independent groups was performed by using unpaired *t*-test for variables with normal distribution and the Wilcoxon rank sum test for the variables that were not normally distributed. The Chi-square test was used for testing the differences in categorical variables between the independent groups. Comparisons within the group (over time) were tested by Friedman test with Dunn’s post test. Results of the continuous variables are expressed as mean ± SD. The changes of blood markers were calculated as post- minus pre-treatment concentrations. The differences were calculated for the first interval after 4 weeks (visit 2–visit 1) and for the whole duration of 8 weeks (visit 3–visit 1). Spearman test was used to study the correlations between different variables.

All statistical tests were two-sided and *p* values <0.05 were considered statistically significant and those between 0.05 and 0.10 were considered to indicate a tendency. Data analyses were performed using SPSS 24.0 and Graph Pad Prism Version 5.04.

## Results

### Baseline characteristics and folate markers

Table 1 shows the main characteristics of the participants according to their treatment allocation. The study groups did not differ in mean age, BMI, creatinine, liver function markers, glucose, or haemoglobin. The prevalence of smoking, use of anti-contraceptive hormones, physical activity, education level, and the number of women with children did not differ between the groups (Table 1).

**Table 1:** Baseline characteristics of the 198 participating women in the 400 and 800 µg/day group

	400 µg/day *n* = 99	800 µg/day *n* = 99	*p*
Age, years	26.6 ± 5.7	26.9 ± 7.1	0.617^a^
BMI, kg/m^2^	21.9 ± 2.8	22.0 ± 2.6	0.718^a^
Creatinine, µmol/L	68.1 ± 8.0	68.1 ± 10.6	0.957^b^
ALT, U/L	18.4 ± 7.7	17.4 ± 7.9	0.348^a^
AST, U/L	18.6 ± 6.4	18.8 ± 6.4	0.960^a^
Fasting glucose, mmol/L	4.6 ± 0.4	4.6 ± 0.3	0.296^b^
Hemoglobin, g/dL	13.3 ± 0.9	13.1 ± 0.9	0.102^b^
Hematocrit, %	39.9 ± 2.5	39.2 ± 2.6	0.056^b^
Mean corpuscular volume, fl	87.5 ± 4.1	87.4 ± 4.6	0.805^a^
Smoking (yes), *n* (%)	19 (19.2)	14 (14.1)	0.340^c^
Use of anti-contraceptive hormones (yes), *n* (%)	62 (62.6)	58 (58.6)	0.663^c^
Physical activity (yes), *n* (%)	78 (78.8)	75 (75.8)	0.611^c^
Education level, *n* (%)			
Less than university	26 (26.3)	29 (29.3)	0.863^c^
University student	36 (36.4)	33 (33.3)	
University degree or higher	37 (37.4)	37 (37.4)	
Women having children (yes),* n* (%)	15 (15.2)	19 (19.2)	0.451^c^

Results are shown as mean ± SD or number (%)

*ALT* alanine transaminase, *AST* aspartate transaminase, *BMI* body mass index

^a^ *p* values are according to Wilcoxon rank sum test when variables are not normally distributed

^b^ *p* values are according to unpaired *t*-test for normally distributed data

^c^ *p* values are according to Chi-square test

The baseline concentrations of serum folate, RBC-folate, and plasma tHcy did not differ between the study groups (Table 2). Twelve of the 198 participants (6.1%) had RBC-folate in the deficiency range <340 nmol/L, while only 24 (12%) had RBC-folate concentrations ≥906 nmol/L at baseline. At baseline, the percentages of women with folate deficiency (in both groups 6.1%) and those with sufficient RBC-folate (10.1 vs. 14.1%; *p* = 0.384) were comparable in the 400 and 800 µg/day groups. The median alcohol consumption as reported in the 2 × 3 days food protocol was 2.6 g/day, with only five women exceeding 20 g/day. At baseline, no significant differences were found between women with alcohol intake above or below the median in serum folate, RBC-folate, or tHcy (data not shown).

**Table 2:** Concentrations of folate biomarkers in non-pregnant women at visits 1, 2, and 3 and their changes according to treatment allocation

	Visit 1 (baseline)			Visit 2 (4 weeks)			Visit 3 (8 week)		
	400 µg/day	800 µg/day	*p* ^a^	400 µg/day	800 µg/day	*p* ^a^	400 µg/day	800 µg/day	*p* ^a^
Number	99	99		99	99		99	99	
Serum folate, nmol/L	16.7 ± 8.1	18.3 ± 11.9	0.527	37.0 ± 15.8	52.3 ± 18.7	<0.001	45.7 ± 16.9	67.2 ± 19.8	<0.001
RBC-folate, nmol/L	632 ± 285	652 ± 295	0.578	805 ± 363	928 ± 330	<0.001	1021 ± 414	1218 ± 435	<0.001
tHcy, µmol/L	7.6 ± 2.1	7.5 ± 2.8	0.347	5.7 ± 1.5	5.7 ± 1.4	0.043	5.9 ± 1.4	5.6 ± 1.3	0.123
RBC/serum folate ratio	41.4 ± 17.6	40.7 ± 18.3	0.509	24.5 ± 11.9	20.0 ± 11.1	0.001	24.2 ± 10.2	20.1 ± 11.6	<0.001
RBC-folate ≥906 nmol/L, *n* (%)	10 (10.1%)	14 (14.1%)	0.384^b^	31 (31.3%)	45 (45.5%)	0.041^b^	54 (54.5%)	83 (83.8%)	<0.001^b^
RBC-folate <340 nmol/L, *n* (%)	6 (6.1%)	6 (6.1%)	0.999^b^	1 (1.0%)	0 (0%)	–	–	–	–

				Changes from visit 1 to 2 (4 weeks)^c^			Changes from visit 1 to 3 (8 weeks)^c^		
				400 µg/day	800 µg/day	*p* ^a^	400 µg/day	800 µg/day	*p* ^a^
Serum folate, nmol/L				20.2 ± 11.8	33.9 ± 16.2	<0.001	29.0 ± 13.6	48.9 ± 17.4	<0.001
RBC-folate, nmol/L				173 ± 174	276 ± 157	<0.001	389 ± 229	566 ± 260	<0.001
tHcy, µmol/L				−1.5 ± 1.3	−1.8 ± 2.2	0.314	−1.7 ± 1.4	−1.9 ± 2.3	0.757

Results are shown as mean ± SD

*RBC* red blood cell, *tHcy* total homocysteine

^a^ *p* values are according to Wilcoxon rank sum test (continuous variables)

^b^ *p* values are according to Chi-square test

^c^Changes are calculated as concentrations at 4 weeks (or 8 weeks) minus those at baseline

### Post intervention folate markers and their changes

After 4 weeks, the 800 µg/day group had significantly higher serum folate (mean = 52.3 vs. 37.0 nmol/L; *p* < 0.001) and RBC-folate (928 vs. 805 nmol/L; *p* < 0.001) than the 400 µg/day group (Table 2). The percentage of women who achieved RBC-folate concentrations in the desirable range (≥906 nmol/L) after 4 weeks was significantly higher in the 800 µg/day group compared with the 400 µg/day group (45.5 vs. 31.3%; *p* = 0.041). The ratio of RBC-folate/serum folate declined strongly in both study groups after 4 weeks of intervention with folate, but it was lower in the 800 µg/day group compared with the 400 µg/day group after 4 weeks (20.0 vs. 24.5; *p* < 0.001). The plasma concentrations of tHcy were lower in the 800 µg/day compared with the 400 µg/day group after 4 weeks (mean = 5.7 vs. 6.1 µmol/L, *p* = 0.043) (Table 2).

The concentrations of serum and RBC-folate continued to increase between 4 and 8 weeks, but remained higher in the 800 µg/day group as compared with the 400 µg/day group after 8 weeks (serum folate = 67.2 vs. 45.7 nmol/L; RBC-folate = 1218 vs. 1021 nmol/L; both *p* < 0.001). The ratio of RBC-folate/serum folate did not show any further changes from 4 to 8 weeks, suggesting that RBC-folate increased parallel to the increase of serum folate after the first 4 weeks. At 8 weeks, significantly more women in the 800 µg/day group achieved desirable RBC-folate concentrations compared with the 400 µg/day group (83.8 vs. 54.5%; *p* < 0.001). The difference in plasma tHcy between the groups was not significant after 8 weeks (5.6 vs. 5.9 µmol/L, in the 800 and 400 µg/day, respectively; *p* = 0.123) (Table 2).

The changes of serum folate after 4 weeks (mean = +33.9 vs. +20.2 nmol/L; *p* < 0.001) and 8 weeks (+48.9 vs. +29.0 nmol/L; *p* < 0.001) were significantly higher in the 800 µg/day group compared with the 400 µg/day. Similarly, the changes of RBC-folate after 4 weeks (+276 vs. +173 nmol/L) and 8 weeks (+566 vs. +389 nmol/L) were higher in the 800 µg/day (both *p* < 0.001). Plasma tHcy declined in both groups (−1.7 and −1.9 µmol/L in the 400 and 800 µg/day after 8 weeks of supplementation), but the changes were not significantly different between the groups (Table 2).

Supplemental Fig. 2 shows mean (95% confidence intervals, CI) of concentrations of serum folate, RBC-folate and tHcy according to the intervention and the duration of the treatment. The longitudinal changes of all markers from baseline to visit 2 or 3 were significant in both groups.

### Changes of folate markers according to baseline RBC-folate

We studied the differences in folate markers and their changes between the study groups according to baseline RBC-folate (<, ≥590 nmol/L, the median of the study population) (Tables 3, 4). Women with low baseline RBC-folate had approximately 50% lower serum folate compared with women with RBC-folate ≥590 nmol/L. Mean baseline plasma tHcy was low in both subgroups but tended to be lower in the subgroup with RBC-folate concentrations ≥590 nmol/L compared with the group with RBC-folate below this limit (7.2 vs. 7.9 µmol/L, respectively; *p* = 0.098). tHcy was lowered after supplementation, but the changes were not different between the study arms (Tables 3, 4).

**Table 3:** Changes of the concentrations of folate biomarkers within 4 and 8 weeks according to treatment allocation in the subgroup of women with baseline RBC-folate concentrations <590 nmol/L

	Visit 1 (baseline)			Visit 2 (4 weeks)			Visit 3 (8 week)		
	400 µg/day	800 µg/day	*p* ^*a*^	400 µg/day	800 µg/day	*p* ^a^	400 µg/day	800 µg/day	*p* ^a^
Numbers	50	49		50	49		50	49	
Serum folate, nmol/L	12.7 ± 4.7	12.6 ± 4.1	0.561	31.0 ± 9.5	44.3 ± 15.6	0.001	39.9 ± 12.5	59.9 ± 18.8	<0.001
RBC-folate, nmol/L	435 ± 95	447 ± 90	0.385	598 ± 114	740 ± 141	<0.001	778 ± 139	1007 ± 166	<0.001
tHcy, µmol/L	7.9 ± 2.3	7.9 ± 3.4	0.493	6.1 ± 1.5	5.7 ± 1.4	0.125	5.8 ± 1.5	5.5 ± 1.3	0.166
RBC/serum folate ratio	38.0 ± 14.5	38.1 ± 11.5	0.721	21.4 ± 8.6	19.9 ± 13.5	0.057	21.6 ± 8.4	19.3 ± 12.3	0.040
RBC-folate ≥906 nmol/L, *n* (%)	0	0	–	0	5 (10.2%)	0.020^b^	10 (20.0%)	34 (69.4%)	<0.001^b^

				Changes from visit 1 to 2 (4 weeks)^c^			Changes from visit 1 to 3 (8 weeks)^c^		
				400 µg/day	800 µg/day	*p* ^a^	400 µg/day	800 µg/day	*p* ^a^
Serum folate, nmol/L				18.4 ± 8.6	31.7 ± 14.0	<0.001	27.3 ± 12.2	47.3 ± 18.6	<0.001
RBC-folate, nmol/L				163 ± 95	293 ± 111	<0.001	343 ± 135	561 ± 135	<0.001
tHcy, µmol/L				−1.7 ± 1.4	−2.2 ± 2.8	0.588	−2.1 ± 1.5	−2.4 ± 2.9	0.903

Results are shown as mean ± SD

Median baseline RBC-folate in the whole study population = 590 nmol/L

*RBC* red blood cell, *tHcy* total homocysteine

^a^ *p* values are according to Wilcoxon rank sum test (continuous variables)

^b^ *p* values are according to Chi-square test

^c^Changes are calculated as concentrations at 4 weeks (or 8 weeks) minus those at baseline

**Table 4:** Changes of the concentrations of folate biomarkers within 4 and 8 weeks according to treatment allocation in the subgroup of women with baseline RBC-folate concentrations ≥590 nmol/L

	Visit 1 (baseline)			Visit 2 (4 weeks)			Visit 3 (8 week)		
	400 µg/day	800 µg/day	*p* ^b^	400 µg/day	800 µg/day	*p* ^b^	400 µg/day	800 µg/day	*p* ^b^
Number	49	50		49	50		49	50	
Serum folate, nmol/L	20.9 ± 8.8	23.9 ± 14.2	0.573	43.1 ± 18.6	60.1 ± 18.3	<0.001	51.6 ± 18.8	74.4 ± 18.1	<0.001
RBC-folate, nmol/L	833 ± 273	853 ± 287	0.721	1017 ± 407	1112 ± 358	0.020	1268 ± 454	1425 ± 513	0.019
tHcy, µmol/L	7.3 ± 1.8	7.0 ± 1.9	0.515	6.1 ± 1.4	5.7 ± 1.4	0.134	5.9 ± 1.4	5.6 ± 1.3	0.421
RBC/serum folate ratio	44.9 ± 20.0	43.2 ± 23.0	0.240	27.5 ± 13.9	20.1 ± 8.1	0.004	27.0 ± 11.3	20.8 ± 11.0	<0.001
RBC-folate ≥906 nmol/L, *n* (%)	10 (20.4%)	14 (28.0%)	0.378^c^	31 (63.3%)	40 (80%)	0.065^c^	44 (89.8%)	49 (98%)	0.087^c^

				Changes from visit 1 to 2 (4 weeks)^d^			Changes from visit 1 to 3 (8 weeks)^d^		
				400 µg/day	800 µg/day	*p* ^b^	400 µg/day	800 µg/day	*p* ^b^
Serum folate, nmol/L				22.1 ± 14.3	36.1 ± 17.9	<0.001	30.7 ± 14.7	50.5 ± 16.2	<0.001
RBC-folate, nmol/L				184 ± 229	259 ± 191	0.022	436 ± 289	572 ± 343	0.005
tHcy, µmol/L				−1.2 ± 1.1	−1.4 ± 1.3	0.497	−1.4 ± 1.2	−1.4 ± 1.3	0.726

Results are shown as mean ± SD

*RBC* red blood cell, *tHcy* total homocysteine

^a^Median baseline RBC-folate in the whole study population = 590 nmol/L

^b^ *p* values are according to Wilcoxon rank sum test (continuous variables)

^c^ *p* values are according to Chi-square test

^d^Changes are calculated as concentrations at 4 weeks (or 8 weeks) minus those at baseline

**Fig. 1:**
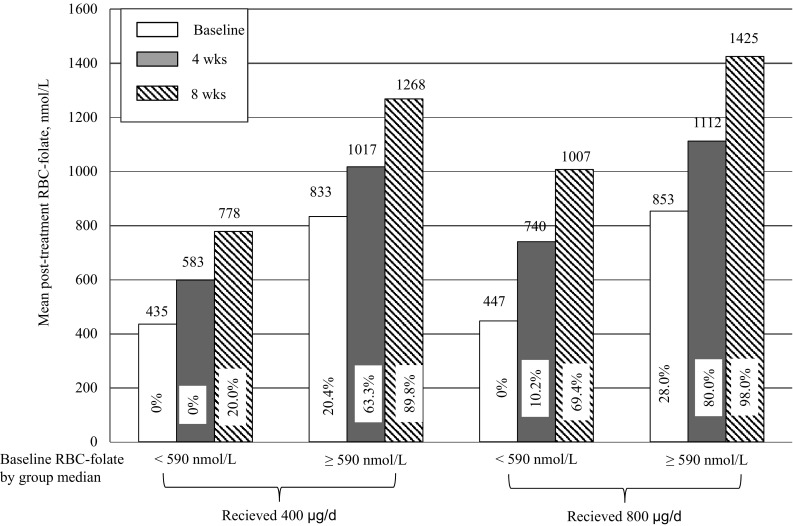
Mean concentrations of RBC-folate at baseline, 4 and 8 weeks after supplementation with 400 and 800 µg/day folate according to baseline RBC-folate (<, ≥group median 590 nmol/L). The percentages within the column indicate the % of women reaching RBC-folate levels ≥906 nmol/L in the subgroups

Women who started the trial with baseline RBC-folate levels <590 nmol/L (Fig. 1; Table 3), maintained lower levels until week 8 as compared with women who started with levels ≥590 nmol/L (Table 4). However, women with low baseline RBC-folate levels who received 800 µg/day for 8 weeks were more likely to achieve the desirable range compared with those in the 400 µg/day group (69.4 vs. 20.0%) (Table 3). Women with low RBC-folate at start had reached a mean RBC-folate of 598 nmol/L (none ≥906 nmol/L) after 4 weeks and a mean of 778 nmol/L (20%, ≥906 nmol/L) after 8 weeks (Fig. 1). In the subgroup of women with RBC-folate ≥590 nmol/L, 80% of the participants reached desirable folate concentrations within 4 weeks of supplementing 800 µg/day, while 63.3% reached similar RBC folate range within 4 weeks of supplementation with 400 µg/day (Table 4, Fig. 1).

In the 400 µg/day group, there was no significant correlation between baseline RBC-folate and delta RBC-folate at 4 weeks (Spearman correlation *r* = −0.05, *p* = 0.641) or that at 8 weeks (*r* = 0.04, *p* = 0.670). The changes of RBC-folate in the 800 µg/day group showed a weak, but significant negative correlation with baseline RBC-folate at 4 weeks (*r* = −0.22, *p* = 0.033), but not at 8 weeks (*r* = −0.10, *p* = 0.318). Thus, the supplemental dose and time on supplements had a stronger influence on the changes of RBC-folate compared with the influence of baseline RBC-folate concentrations.

### RBC-folate as a function of serum folate under supplementation

The post-intervention concentrations of RBC-folate were stratified into ranges that differ by 100 nmol/L (Supplemental Figs. 3 and 4). The response of serum concentrations of folate to treatment was strong and concentrations were not proportional to RBC-folate levels after supplementation. At 8 weeks, serum folate ≥47.0 nmol/L in the 400 µg/day or ≥61.2 nmol/L in the 800 µg/day group corresponded to RBC-folate concentrations of >940 nmol/L.

Ten intolerance cases were documented and considered related to the study supplements (mostly gastrointestinal intolerability), but in only one case, the participant dropped out (in the 400 µg/day group). No serious adverse events (including no death) were reported and no differences in reported adverse events between the study groups.

## Discussion

Eliminating folate deficiency is not sufficient to reduce the risk of birth defects. A population optimal RBC-folate of ≥906 nmol/L for defining folate sufficiency in young women is not achievable in countries not applying fortification. The present study has shown that 6.1% of the participating women would be considered deficient according to the classical definition (RBC-folate <340 nmol/L), while 88% had baseline RBC-folate concentrations <906 nmol/L that are considered associated with a high risk of NTDs. The majority of the women from this population can be expected to have insufficient pre-conceptional folate status, unless supplements have been used in an appropriately high dose and for approximately 8 weeks. Approximately, 45.5% of all women who received 400 µg/day did not achieve RBC-folate ≥906 nmol/L after 8 weeks, while this portion was 80% in the women with RBC-folate levels below the population median (590 nmol/L) who received 400 µg/day for 8 weeks. When 800 µg/day folate was supplemented, only 16.2% of the women did not achieve the target RBC-folate levels after 8 weeks. In both study arms, women who started the trial with a low baseline RBC-folate (<median of the population) maintained lower concentrations compared with those who started with higher levels. In a country without folic acid fortification, the current recommendation to supplement 400 µg/day is not sufficient to achieve protective RBC-folate levels if supplemented for 4–8 weeks.

### The relationship between serum and RBC-folate under supplementation

Serum folate is thought to be available for transport to the placenta [20]. Using RBC-folate for screening purposes in women is recommended, but currently not widely practiced. Daly et al., suggested that compared with serum folate, RBC-folate can better reflect the risk of NTDs in the first trimester of pregnancy [12, 17]. The concentrations of serum folate markedly increased in all women within the first 4 weeks after supplementation, but many of the participants (especially in the 400 µg/day group) did not achieve a desirable RBC-folate. The changes of serum folate were stronger compared with rather limited changes of RBC-folate in the same individuals. Raising RBC-folate requires incorporating folate into newly formed RBCs which requires longer duration and is a rate-limiting step in women with low baseline RBC-folate. An intriguing question is whether similar rate limiting steps exist regarding folate transfer to the placenta and foetus.

### Practical implications and impact on future studies and NTD prevention

The average concentrations of RBC-folate in the current study are comparable with earlier studies in young women from the same population (i.e., 500–600 nmol/L) [14, 15, 21]. Similar levels were reported in women from other European countries (i.e., 460 nmol/L in Dutch, 535 nmol/L in UK, and 468 nmol/L in Irish women) [22, 23], although levels tended to be higher in adults from countries with widespread voluntary fortifications (i.e., Ireland) [9].

An important question arises concerning the expected magnitude of NTD risk reduction after improving RBC-folate. According to the estimated NTD prevalence data (per 10,000 births) in Germany over 11 years from 2000 to 2010 [mean (95% confidence intervals) = 10.76 (9.45–12.19)] [10] and considering the risk reduction models by Crider et al., [13], we estimate that increasing RBC-folate from an average of 600 to 1000 nmol/L or 1200 nmol/L would reduce the NTD risk to 7.9 or 5.8 per 10,000 births, respectively. However, although it can be inferred from the data, the current study does not provide evidence that 800 µg/day can prevent more NTDs cases than 400 µg/day if both doses would be taken for the same duration. Obviously, the role of folate in NTD prevention depends on the time available to increase blood folate before the closure of the neural tube in the first trimester.

As in earlier studies in this population [14, 15, 21], our study included relatively educated women and our exclusion criteria are likely to restrict the number of women with folate deficiency. Nevertheless, only 12% of the participants in the current study had concentrations of RBC-folate in the optimal range. We expect that the population average of RBC-folate is even below the median of the current study (590 nmol/L). The recommended RBC-folate ≥906 nmol/L is not thought to predict the risk reduction on the individual level. However, on a population level, a typical western diet is unlikely to provide the intake amount necessary to achieve these high RBC-folate levels. Most fortification programs provide approximately 150 µg folic acid/day, but still 30–40% of adults men and women can have RBC-folate below 906 nmol/L after the fortification [24]. Even in the post fortification era, folic acid supplement use was associated with a RBC-folate concentration ≥906 nmol/L [25]. Over 50% of our participants did not reach a desirable RBC-folate within 4–8 weeks on 400 µg/day. Thus, if a pregnancy may occur within weeks, 800 µg/day appears to be more appropriate, in particular for women with low RBC-folate or in countries not applying fortification with folic acid.

### Study limitations

The study has limitations that deserve mentioning. The design (i.e., open-labelled and non-placebo controlled) could be seen as a limitation. However, since the participants were rather educated and showed high compliance, we judged it as unlikely that women would have consumed additional supplements during the study. The changes and differences in blood markers between the groups and the comparability with earlier studies confirm this assumption. Moreover, the relatively short duration does not allow reaching a steady state for RBC-folate. However, the 8 weeks were judged based on current recommendations and a realistic time window for women planning pregnancy.

In summary, we have shown that 6.1% of German women in pregnancy age are considered folate deficient, but 88% have insufficient RBC-folate to protect against NTD in the event of pregnancy. Serum and RBC-folate concentrations increased after supplementing 400 µg/day folate for 4–8 weeks, but the majority of women maintained RBC-folate concentrations below 906 nmol/L. The higher dose of folate (800 µg/day) appears to be more appropriate than the 400 g/day when folate status has to be improved within 4–8 weeks, in particular for women with RBC-folate below the population average. The impact of the difference in the response to different doses of folate on the risk of NTDs and the number of NTD-affected pregnancies deserve further investigations.

## Electronic supplementary material

Below is the link to the electronic supplementary material.

Supplementary material 1 (PPTX 116 kb)

Supplementary material 2 (DOCX 23 kb)

## Abbreviations

ALT: Alanine transaminase
AST: Aspartate transaminase
BMI: Body mass index
(6S)-5-CH_3_-H_4_folate-Ca: (6S)-5-methyltetrahydrofolate calcium
RBC: Red blood cells
NTD: Neural tube defect
RDA: Recommended dietary allowance
tHcy: Total homocysteine
WHO: World Health Organisation
