# Supplementary material for: The effectiveness of daily supplementation with 400 or 800 µg/day folate in reaching protective red blood folate concentrations in non-pregnant women: a randomized trial
Source: Eur J Nutr. 2017 Apr 26;57(5):1771–80. doi: 10.1007/s00394-017-1461-8 (PMC6060806; doi:10.1007/s00394-017-1461-8)
Supplement: Supplementary file 1 — Supplementary material 1 (PPTX 116 kb) [file 394_2017_1461_MOESM1_ESM.pptx]

## Slide 1
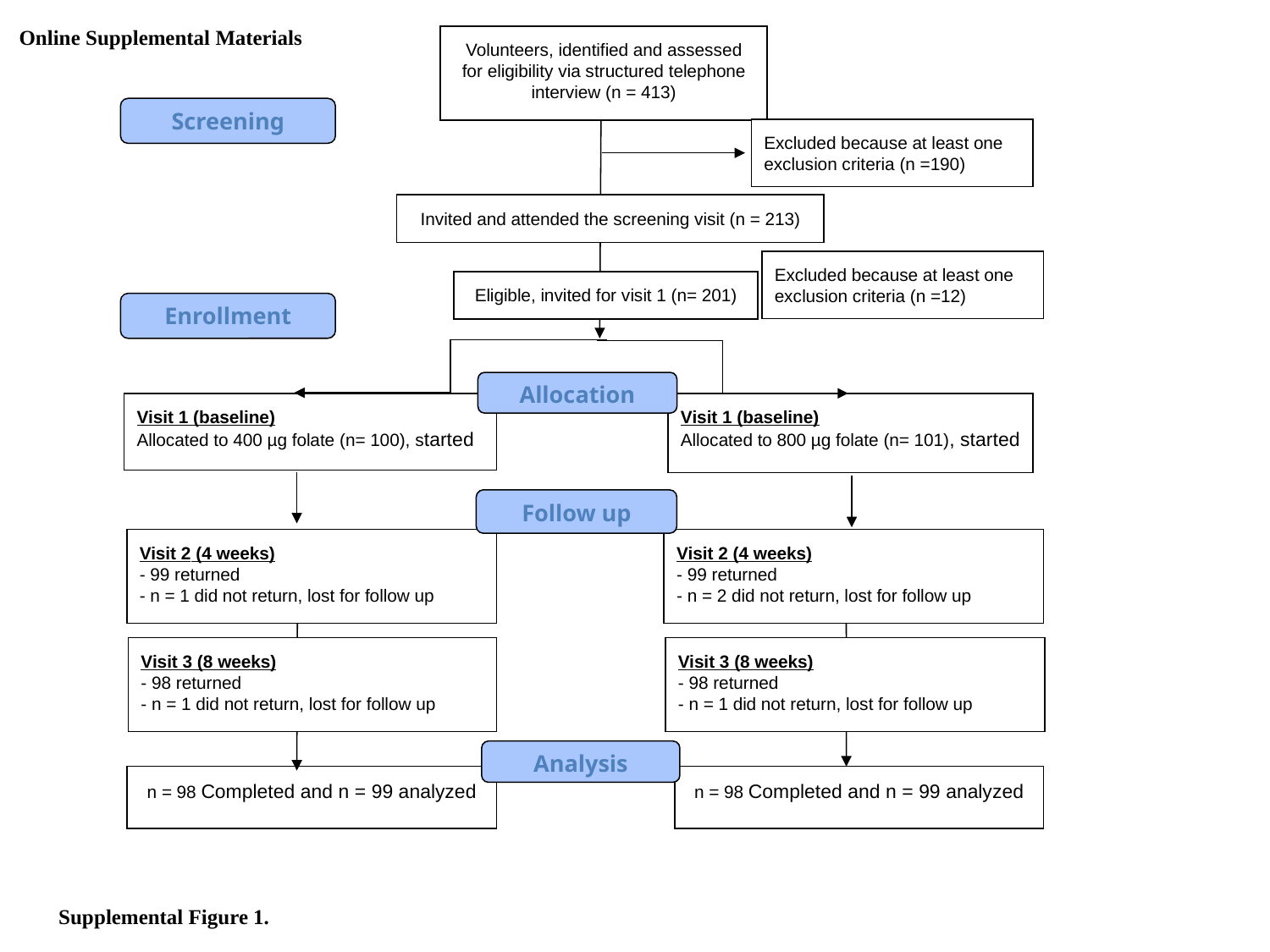

Online Supplemental Materials
Volunteers, identified and assessed for eligibility via structured telephone interview (n = 413)
Screening
Excluded because at least one exclusion criteria (n =190)
Invited and attended the screening visit (n = 213)
Excluded because at least one exclusion criteria (n =12)
Eligible, invited for visit 1 (n= 201)
Enrollment
Allocation
Visit 1 (baseline)
Allocated to 800 µg folate (n= 101), started
Visit 1 (baseline)
Allocated to 400 µg folate (n= 100), started
Follow up
Visit 2 (4 weeks)
- 99 returned
- n = 2 did not return, lost for follow up
Visit 2 (4 weeks)
- 99 returned
- n = 1 did not return, lost for follow up
Visit 3 (8 weeks)
- 98 returned
- n = 1 did not return, lost for follow up
Visit 3 (8 weeks)
- 98 returned
- n = 1 did not return, lost for follow up
Analysis
n = 98 Completed and n = 99 analyzed
n = 98 Completed and n = 99 analyzed
Supplemental Figure 1.

## Slide 2
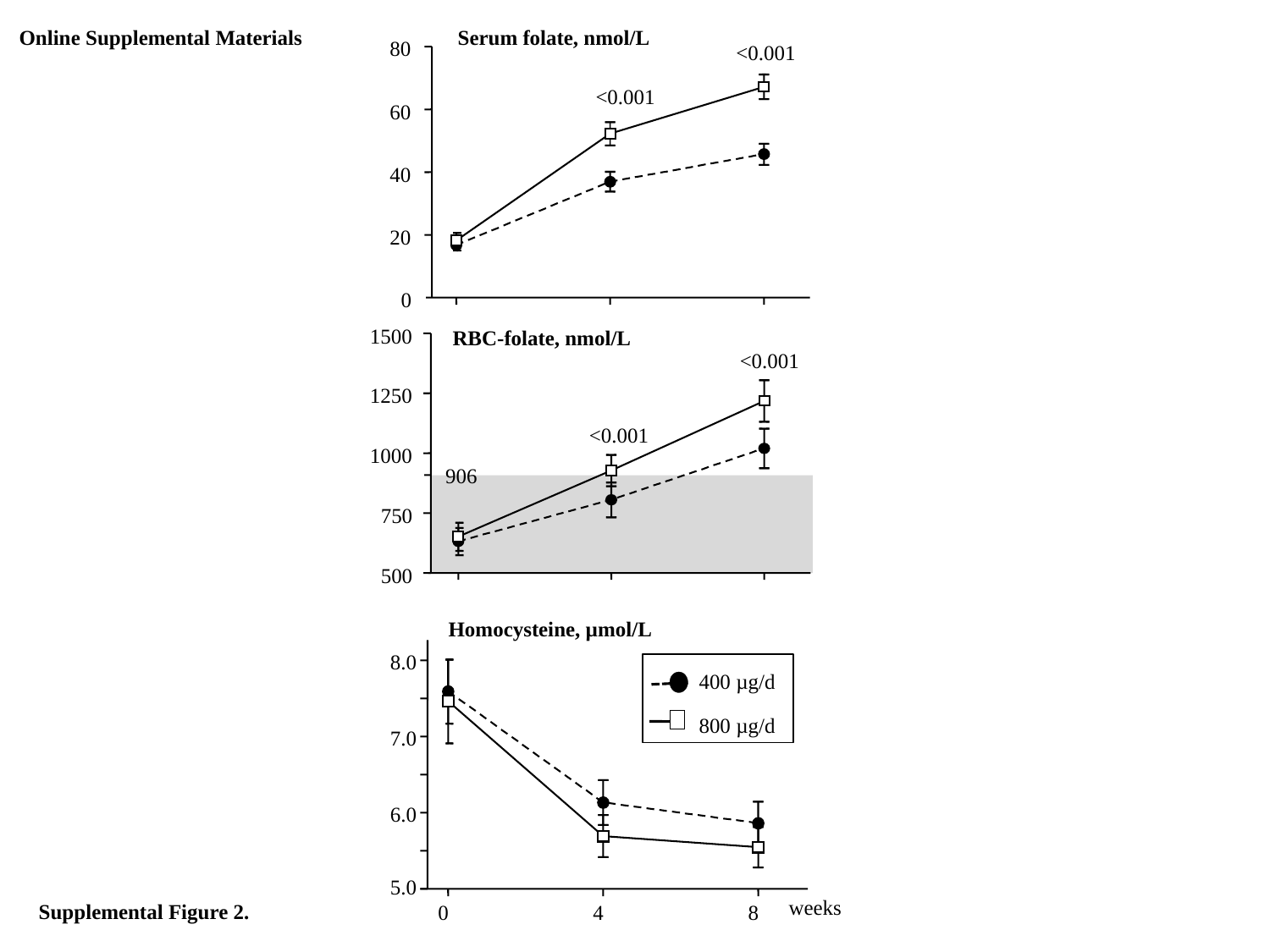

Online Supplemental Materials
Serum folate, nmol/L
80
<0.001
<0.001
60
40
20
0
1500
RBC-folate, nmol/L
<0.001
1250
<0.001
1000
906
750
500
Homocysteine, µmol/L
8.0
400 µg/d
800 µg/d
7.0
6.0
5.0
weeks
0
4
8
Supplemental Figure 2.

## Slide 3
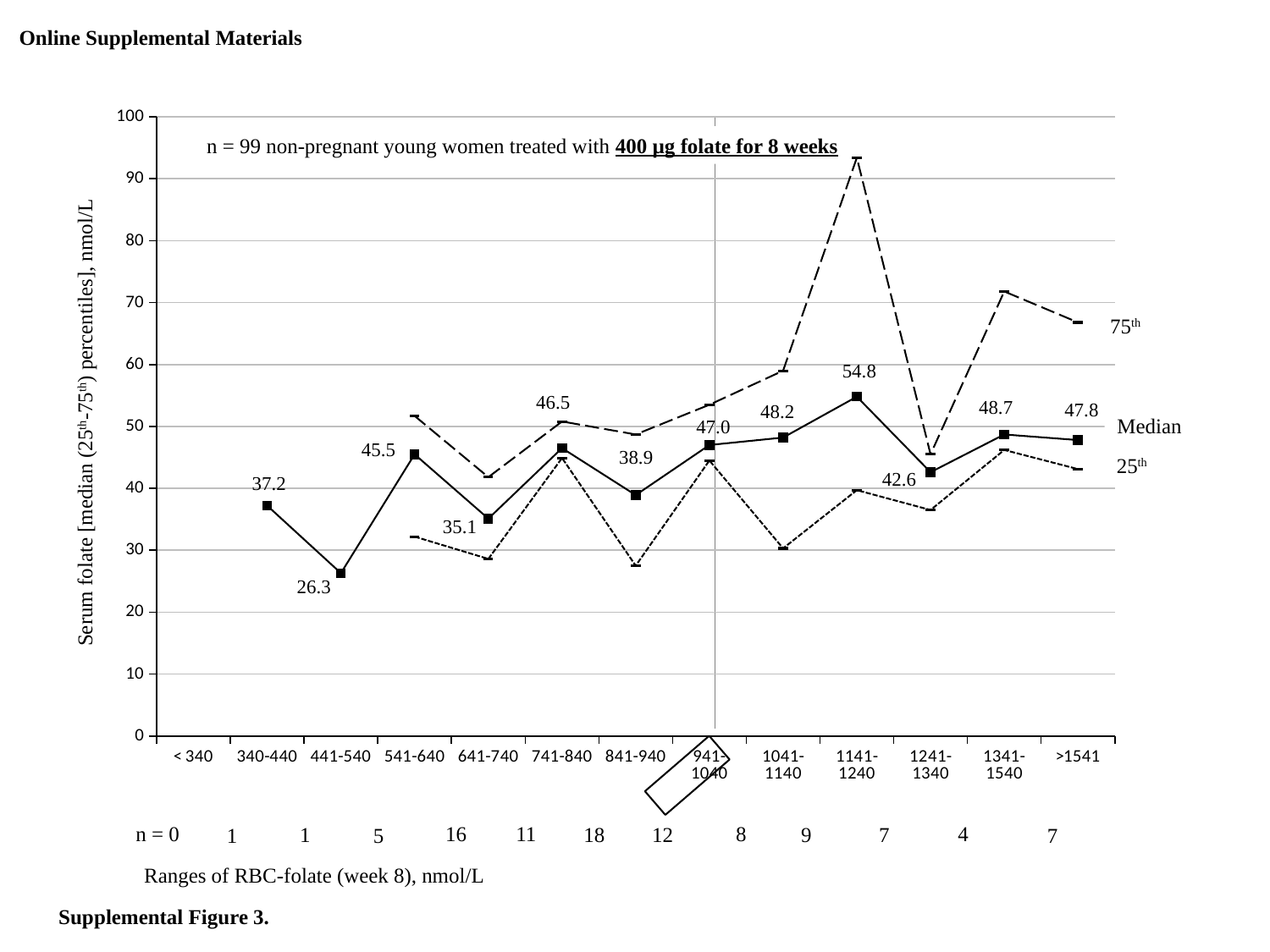

Online Supplemental Materials
### Chart
| Category | Median | 25th | 75th |
|---|---|---|---|
| < 340 | None | None | None |
| 340-440 | 37.2 | None | None |
| 441-540 | 26.3 | None | None |
| 541-640 | 45.5 | 32.2 | 51.7 |
| 641-740 | 35.1 | 28.6 | 41.8 |
| 741-840 | 46.5 | 44.9 | 50.8 |
| 841-940 | 38.9 | 27.5 | 48.7 |
| 941-1040 | 47.0 | 44.5 | 53.5 |
| 1041-1140 | 48.2 | 30.3 | 59.0 |
| 1141-1240 | 54.8 | 39.7 | 93.4 |
| 1241-1340 | 42.6 | 36.5 | 45.5 |
| 1341-1540 | 48.7 | 46.2 | 71.8 |
| >1541 | 47.8 | 43.1 | 66.8 |n = 99 non-pregnant young women treated with 400 µg folate for 8 weeks
75th
54.8
46.5
48.7
47.8
48.2
Serum folate [median (25th-75th) percentiles], nmol/L
Median
47.0
45.5
38.9
25th
42.6
37.2
35.1
26.3
11
n = 0
16
8
4
1
18
12
9
7
1
5
7
Ranges of RBC-folate (week 8), nmol/L
Supplemental Figure 3.

## Slide 4
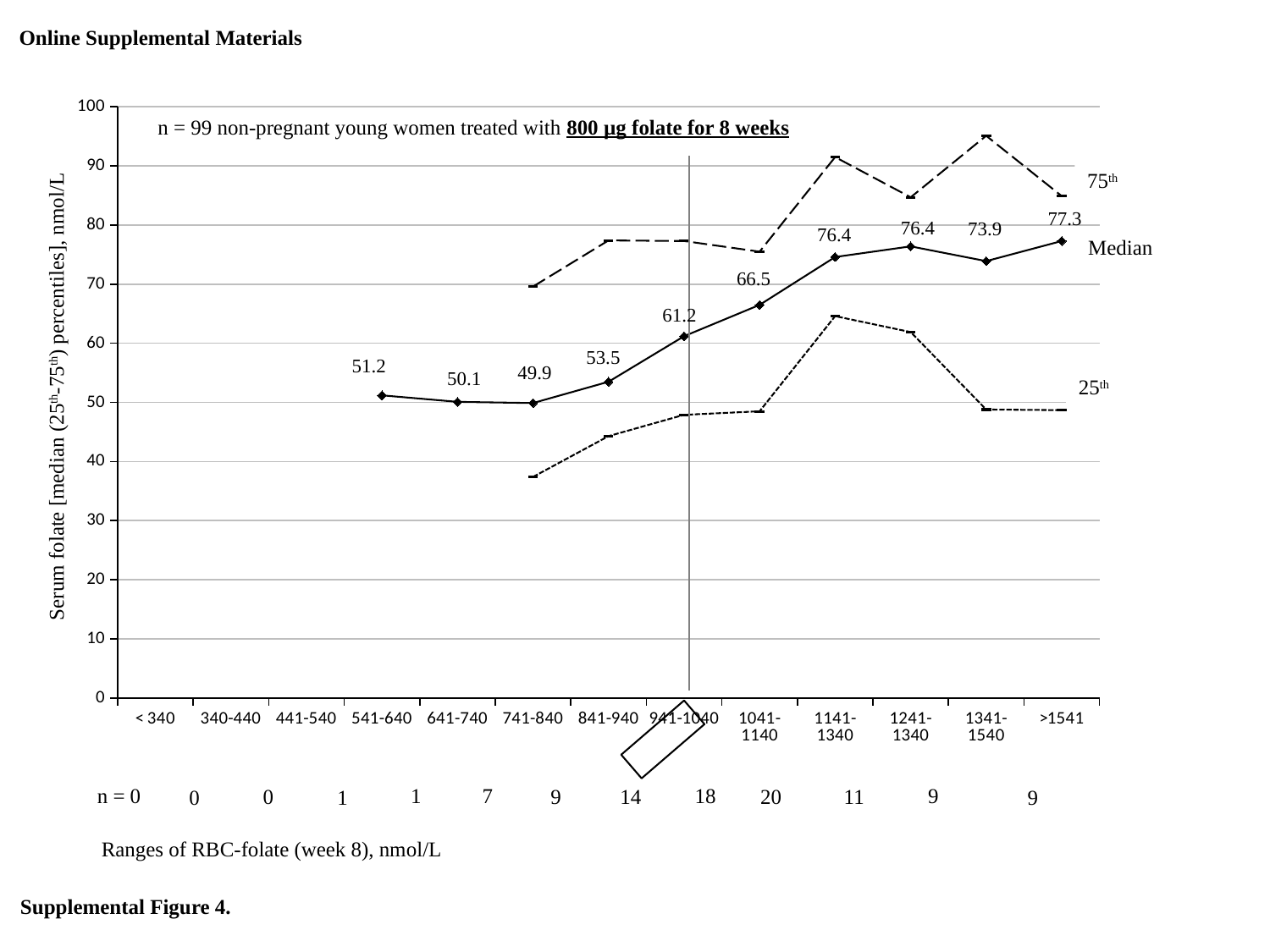

Online Supplemental Materials
### Chart
| Category | Median | 25th | 75th |
|---|---|---|---|
| < 340 | None | None | None |
| 340-440 | None | None | None |
| 441-540 | None | None | None |
| 541-640 | 51.2 | None | None |
| 641-740 | 50.1 | None | None |
| 741-840 | 49.9 | 37.4 | 69.6 |
| 841-940 | 53.5 | 44.3 | 77.4 |
| 941-1040 | 61.2 | 47.9 | 77.3 |
| 1041-1140 | 66.5 | 48.5 | 75.5 |
| 1141-1340 | 74.6 | 64.6 | 91.5 |
| 1241-1340 | 76.4 | 61.9 | 84.7 |
| 1341-1540 | 73.9 | 48.8 | 95.1 |
| >1541 | 77.3 | 48.7 | 84.9 |n = 99 non-pregnant young women treated with 800 µg folate for 8 weeks
75th
77.3
76.4
73.9
76.4
Median
66.5
61.2
53.5
51.2
49.9
50.1
25th
Serum folate [median (25th-75th) percentiles], nmol/L
7
n = 0
1
18
9
0
9
14
20
11
9
0
1
Ranges of RBC-folate (week 8), nmol/L
Supplemental Figure 4.
