# Supplementary material for: The effectiveness of daily supplementation with 400 or 800 µg/day folate in reaching protective red blood folate concentrations in non-pregnant women: a randomized trial
Source: Eur J Nutr. 2017 Apr 26;57(5):1771–80. doi: 10.1007/s00394-017-1461-8 (PMC6060806; doi:10.1007/s00394-017-1461-8)
Supplement: Supplementary file 2 — Supplementary material 2 (DOCX 23 kb) [file 394_2017_1461_MOESM2_ESM.docx]

**Supplemental Figure Legends**

**Supplemental Figure 1.** Study Flow Diagram showing the number of participants from the screening phases, enrolment, follow up and the completion of the study.

**Supplementary Figure 2.**  Mean ± 95% confidence intervals of serum folate; RBC-folate; and plasma total homocysteine (tHcy) in women treated with 400 or 800 µg/d folate for 4 and 8 weeks. P values between the groups in the figure are according to Wilcoxon rank sum test.

**Supplementary Figure 3.** Concentrations of serum folate [median (25^th^, 75^th^ percentile)] in nmol/L according to red blood cell (RBC)-folate ranges in women who were treated with 400 µg/d folate for 8 weeks. RBC-folate concentrations were divided into ranges that differed by 100 nmol/L.

**Supplementary Figure 4.** Concentrations of serum folate [median (25^th^, 75^th^ percentile)] in nmol/L according to red blood cell (RBC)-folate ranges in women who were treated with 800 µg/d folate for 8 weeks. RBC-folate concentrations were divided into ranges that differed by 100 nmol/L.

| **Supplemental Table 1.** Composition of the multi-nutrient supplements as declared by the manufacturers^1,2^. | | |
| --- | --- | --- |
|  | **Supplement I^1^**  **400 µg** | **Supplement II^2^**  **800 µg** |
| Vitamin B-1, mg | 1.4 | 1.2 |
| Vitamin B-2, mg | 1.4 | 1.6 |
| Vitamin B-6, mg | 1.9 | 1.9 |
| Vitamin B-9 (folate), µg | 400 [folic acid,  (6S)-5-CH_3_-H_4_folate-Ca (1:1)] | 800 [folic acid,  (6S)-5-CH_3_-H_4_folate-Ca (1:1)] |
| Vitamin B-12, µg | 2.6 | 3.5 |
| Biotin, µg | 30 | 60 |
| Niacin, mg | 18 | 15 |
| Pantothenic acid, mg | 6 | 6 |
| Vitamin E, mg | 10 | 13 |
| Vitamin C, mg | 85 | 110 |
| Vitamin D, µg (IU) | 5 (200) | 20 (800) |
| Iodine, µg | 150 | 150 |
| Vitamin A, µg | 770 | - |
| Magnesium, mg | 57 | - |
| Iron, mg | 14 | - |
| Copper, mg | 1.0 | - |
| Zinc, mg | 10 | - |
| Omega-3-fatty acids, mg | 200 | - |
| Selenium, µg | 60 | - |

^1^ Elevit® gynvital; ^2^ Femibion® 1.
